# Spatiotemporal Overlap of Mallards With Poultry Farms Is Associated With Greater Risk of Avian Influenza Wild Bird Spillover Events

**DOI:** 10.1002/ece3.72221

**Published:** 2025-10-01

**Authors:** Joshua A. Cullen, Nicholas M. Masto, Jeffery D. Sullivan, Cory J. Highway, Kelly A. Patyk, Mary‐Jane McCool, Mia Kim Torchetti, Kristina Lantz, Rebecca L. Poulson, Deborah L. Carter, Jamie C. Feddersen, Bradley S. Cohen, Diann J. Prosser

**Affiliations:** ^1^ Eastern Ecological Science Center U.S. Geological Survey Laurel Maryland USA; ^2^ Habitat and Population Evaluation Team U.S. Fish and Wildlife Service Bismark North Dakota USA; ^3^ Animal and Plant Health Inspection Service, Veterinary Services, Strategy and Policy, Center for Epidemiology and Animal Health U.S. Department of Agriculture Fort Collins Colorado USA; ^4^ Animal and Plant Health Inspection Service, Veterinary Services, National Centers for Animal Health, National Veterinary Services Laboratories U.S. Department of Agriculture Ames Iowa USA; ^5^ Southeastern Cooperative Wildlife Disease Study, College of Veterinary Medicine University of Georgia Athens Georgia USA; ^6^ Tennessee Wildlife Resources Agency Nashville Tennessee USA

**Keywords:** animal movement, avian influenza, infectious disease, poultry, spillover events, waterfowl

## Abstract

Animal movement influences local transmission and geographic spread of pathogens. Waterfowl are known reservoirs of pathogens, including H5 goose/Guangdong lineage (H5 GsGd) highly pathogenic avian influenza (HPAI). This HPAI virus lineage causes high rates of morbidity and mortality in domestic poultry and many wild bird species. Mallards ( *Anas platyrhynchos* ) are a generalist waterfowl species whose habitat largely overlaps with many other waterfowl and are considered effective spillover vectors of HPAI. To investigate the potential contribution of waterfowl to HPAI spillover, we used mallards as a proxy and measured the spatiotemporal overlap of 183 GPS‐tagged mallards during 2021–2022 with respect to confirmed wild bird spillover events in United States (U.S.) poultry farms. Additionally, we estimated the probability of HPAI spillover events as a function of mallard overlap and poultry farm type. We found infrequent overlap instances between mallards and poultry farms; however, several of these overlap instances lasted > 5 days and up to 19 days. Population‐level overlap with poultry farms was greatest during pre‐breeding migration, followed by the breeding season. The probability of HPAI spillover was predicted to be greatest for commercial turkey farms, followed by backyard poultry farms. Importantly, farms overlapped by mallards were more than twice as likely to experience a spillover (i.e., increased risk probability), even in the absence of known mallard infection status at the time of overlap. These findings suggest that mallards (and/or other waterfowl) may be important contributors to HPAI spillover into poultry farms and that additional biosecurity measures may be needed. Because few instances of overlap occurred between mallards and farms with reported spillover events, tagged mallards are likely a proxy for other untagged waterfowl. Further studies of wild waterfowl interactions with poultry farms could improve understanding of how landscape characteristics influence spatial overlap, potentially informing which premises may require enhanced biosecurity measures.

## Graphical abstract

GPS‐tagged mallards served as a proxy for wild waterfowl spillover of highly pathogenic avian influenza at domestic poultry farms. Farms overlapped by mallards in space and time were more than twice as likely to experience a spillover event.

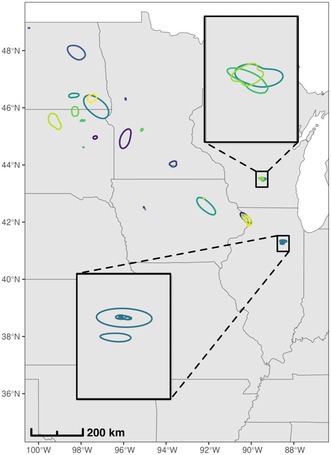

## 1 Introduction

Animal movement is key to the dynamics of local transmission and geographic spread of pathogens (Altizer et al. 2011; Dougherty et al. 2018; Rayl et al. 2021). Movement patterns, contact rates, and species‐habitat associations ultimately affect pathogen exposure and transmission risk (Dougherty et al. 2018; Wilber et al. 2022). For species that exhibit limited dispersal and have highly connected social networks, entire populations may be at risk of rapid pathogen transmission (e.g., Hamede et al. 2009). Alternatively, highly mobile species that travel long distances during seasonal migrations have the potential to spread pathogens from the origin to the destination site, as well as at *en route* stopover locations (Gaidet et al. 2010; Yang et al. 2024). Given the strong association between movement ecology and disease dynamics, the inclusion of animal movement data in disease ecology can provide insights into the potential for pathogen spread and transmission.

Migratory waterfowl (Anatidae) are mobile species that serve as natural reservoirs of pathogens, such as avian influenza virus (AIV) (Olsen et al. 2006). Although low pathogenic avian influenza (LPAI) often circulates with minimal clinical signs in waterfowl, highly pathogenic avian influenza (HPAI) can have dramatic effects on other wild birds and domestic poultry (Swayne 2017). During the recent outbreak of HPAI (goose/Guangdong H5N1 clade 2.3.4.4b) that was confirmed in North America in late 2021 (Bevins et al. 2022), tens of millions of poultry have been infected or culled, and tens of thousands of wild birds have become sick and/or died (Canadian Food Inspection Agency Government of Canada 2024; USDA APHIS 2024a, 2024b; WHISPers 2024). With the continued spread and reassortment of virus subtypes originating from the A/goose/Guangdong/1/1996 lineage (H5 GsGd), this HPAI lineage poses a substantial ongoing threat to wild and domestic birds. Moreover, waterfowl (particularly dabbling ducks (e.g., *Anas*)) are canonical hosts for AIV; therefore, their movements can lead to spillover at the domestic‐wild bird interface despite not necessarily displaying clinical signs of infection (Kim et al. 2009; Prosser et al. 2024; Spackman et al. 2023), which can harm both bird health and agro‐economic interests. Assessing the potential spillover of HPAI into domestic poultry farms that may be attributed to the spatiotemporal movement patterns of wild waterfowl species could help minimize these risks.

Despite the importance of understanding the spread and transmission of HPAI virus, few studies have investigated the potential for transmission at the domestic–wild bird interface. Fewer still have evaluated the spatiotemporal overlap of wild bird movements in relation to poultry farms (e.g., Humphreys et al. 2021; Lee et al. 2020; McDuie et al. 2022, 2024). Collecting data on observed overlap and interactions of waterfowl with poultry farms can enhance understanding of the potential transmission routes at the domestic–wild bird interface, which could be used to mitigate AIV risk via revised biosecurity measures at poultry farms. For example, recent studies have shown AIV detections in North American poultry are positively associated with the occurrence and residence time of migratory dabbling ducks (Humphreys et al. 2020). Because the direct or indirect transmission of AIV is expected to be more likely when wild waterfowl inhabit areas near poultry farms (Mateus‐Anzola et al. 2023; Teitelbaum et al. 2024; Velkers et al. 2021), further evidence could help determine the extent to which overlap by waterfowl is associated with HPAI outbreaks at poultry farms.

Mallards ( *Anas platyrhynchos* ) may serve as an important source of HPAI spillover because they are extremely abundant and widespread, migrate large distances, and occur in areas with high numbers of poultry farms (Teitelbaum et al. 2023). Additionally, mallards often co‐occur in areas occupied by a range of other waterfowl species, and their distributional patterns can serve as a proxy for other wild waterfowl (Casazza et al. 2021; Herbert et al. 2021). Therefore, increasing knowledge of the spatiotemporal patterns of mallard movement with respect to poultry operations may result in better characterization of the risk of HPAI spillover events. The overarching aim of this study was to investigate the extent of mallard spatiotemporal overlap with confirmed HPAI H5N1 detections at poultry farms, which was addressed by (1) estimating observed overlap at the observation and home range levels, (2) predicting overlap at the population level on a seasonal basis, and (3) determining the effects of farm type and mallard overlap with the probability of HPAI H5 GsGd spillover events on poultry farms.

## 2 Materials and Methods

### 2.1 Bird Capture, Tagging, and Sampling

**FIGURE 1:**
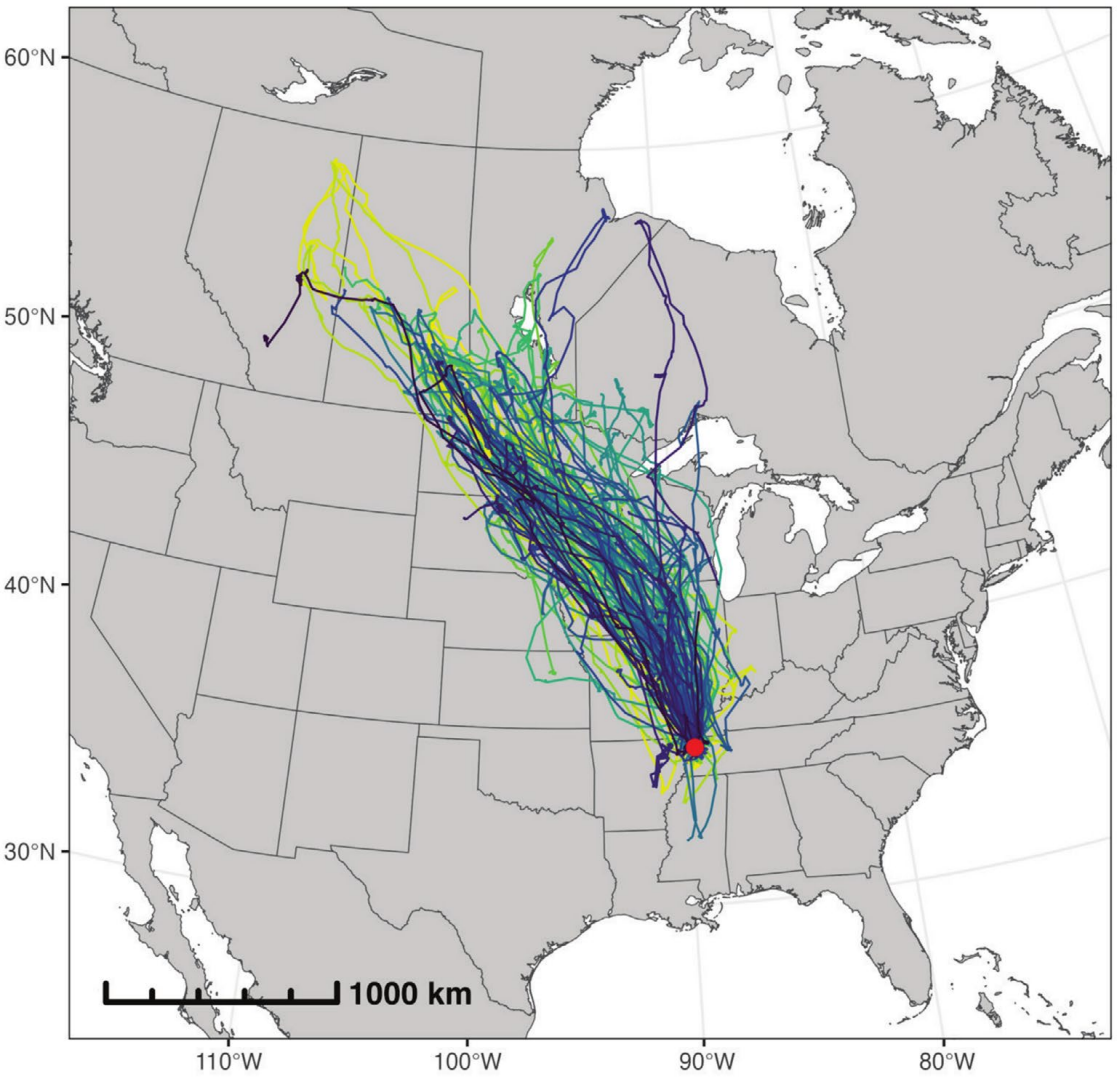
Tracks of 183 mallards (colored lines) tagged in northwest Tennessee during the 2021–2022 sampling period. The red circle denotes the general tagging location. The basemap was created using data from Natural Earth (https://www.naturalearthdata.com).

A total of 183 adult and juvenile mallards were captured using rocket nets, swim‐in traps, and confusion traps at six locations in northwest Tennessee, USA from November 2021 to February 2022 (Figure 1) (Dieter et al. 2009; Sharp and Smith 1986). Mallards were banded, and body mass was measured, where only birds weighing ≥ 1 kg were fitted with 20 g solar rechargeable Global Positioning System‐Global System for Mobile (GPS‐GSM) transmitters (OrniTrack; Ornitela, UAB Švitrigailos, Vilnius, Lithuania) to ensure that the mounted tag remained below recommended body weight limits (3%–5%) (Fair and Jones 2010). These transmitters were attached by dorsally mounting a backpack harness as described in more detail by McDuie et al. (2019). The GPS‐GSM transmitters were remotely programmed to record hourly locations when the battery level was > 75%, every 2 h when the battery level was 25%–75%, and every 6 h when the battery level was < 25% (Masto et al. 2022). Prior calibration of this transmitter model indicated that the median location error was < 25 m (Teitelbaum et al. 2023). All duck capture and handling were conducted in accordance with Tennessee Technological University's Institutional Animal Care and Use Committee (IACUC) protocol #19‐20‐002 and authorized under Federal Banding Permit #05796.

### 2.2 Data Processing and Preparation

**FIGURE 2:**
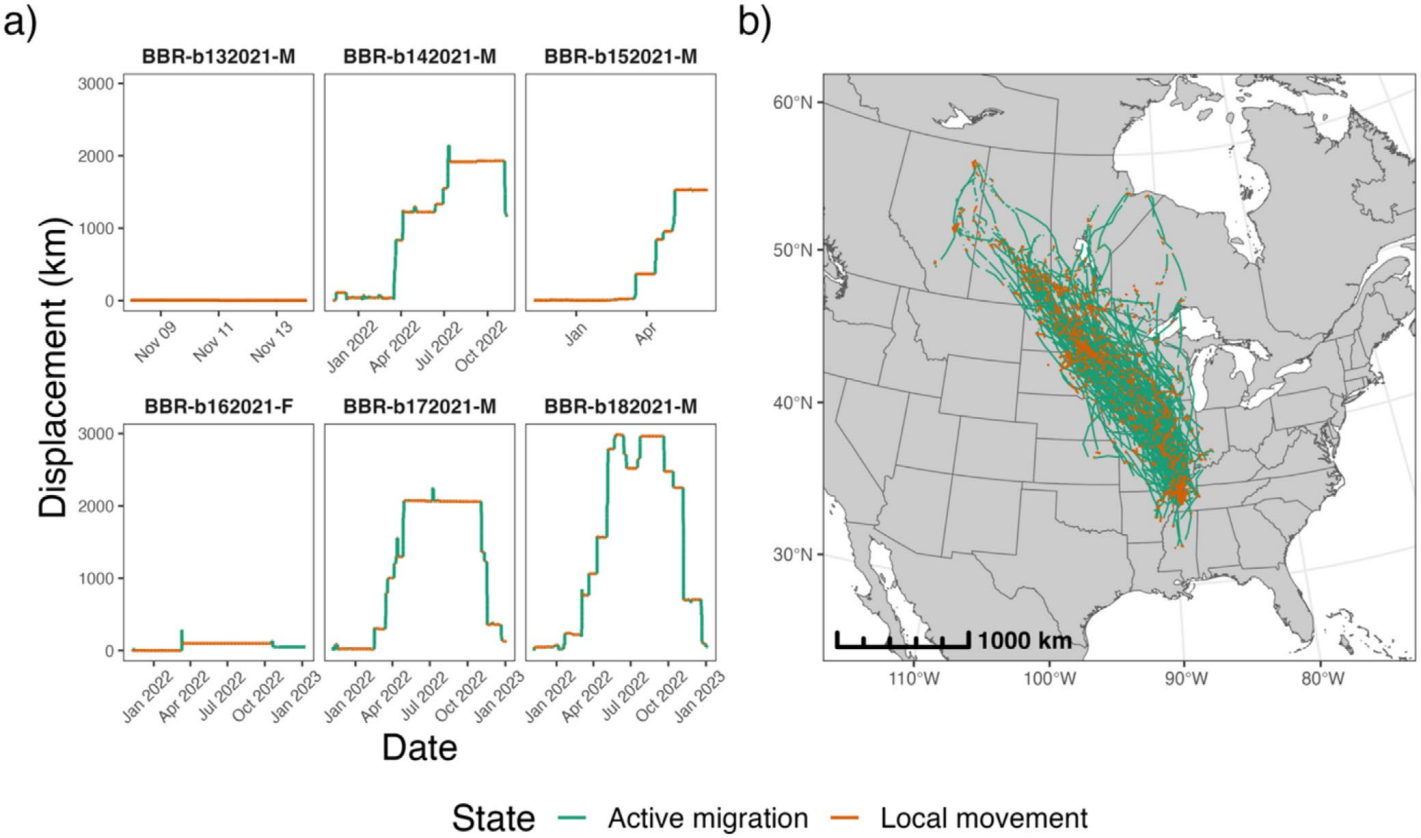
Classified mallard tracks following the segmentation process. (a) Profiles of displacement from the tagging site over time are shown for a subset of six individuals. (b) Classified mallard tracks shown in geographic space. The basemap was created using data from Natural Earth.

For each of the 183 mallard trajectories, step lengths (i.e., the distance between consecutive observations) and displacement (i.e., the distance of every point from the initial location) were quantified to distinguish migratory from range residency (e.g., breeding, wintering, stopover) movement patterns. A thresholding approach for step lengths was initially used to classify tracks into segments of ‘active migration’ and ‘local movement’ behavior, where observations with step lengths > 10 km were classified as ‘active migration’ and smaller step lengths were classified as ‘local movement’. To refine this classification further, the difference in displacement was calculated per new track segment, and the segments with a maximum difference in daily displacement ≥ 5 km were also classified as ‘active migration’. To address anomalously brief segments originally classified as ‘local movement’ but which may not truly represent an individual stopping its migration, track segments classified as ‘local movement’ with < 10 observations or lasting ≤ 1 day were reclassified as ‘active migration’ (Figure 2). This processing step ensured that the results from the simple behavior classification method were improved before proceeding with subsequent steps of data analysis (Hurme et al. 2019; Williams et al. 2017). Following track segmentation, only ‘local movement’ segments were retained for further analyses as these were reflective of extended periods of range‐restricted movements on or near the ground, which would facilitate a greater likelihood of mallards interacting with poultry farms compared to actively migrating birds. This processing step resulted in 1921 ‘local movement’ track segments comprised of 764,839 observations being retained for subsequent steps. All data processing and analyses were conducted using R v4.3.1 (R Core Team 2023).

### 2.3 Space Use Estimation

Segment‐level space use was estimated using autocorrelated kernel density estimation (AKDE) for each ‘local movement’ mallard segment to account for movement when tags were not transmitting (i.e., outside of the 1‐, 2‐, or 6‐h GPS fixes). More specifically, the AKDE method estimates the range distribution (i.e., the long‐term pattern of a movement process that features restricted space use), which serves as a more accurate representation of an animal's home range than traditional methods of kernel density estimation whose assumption of data independence is often violated when using autocorrelated data from modern telemetry devices (Fleming et al. 2015; Silva et al. 2022). First, track segments were each fit to several continuous‐time movement models (CTMMs) to characterize the underlying movement process and estimate autocorrelation (Fleming et al. 2015). From these results, the best‐fitting model (determined via the Akaike information criteria; Akaike 1973) was retained for space use estimation via AKDE using the *ctmm* R package (Calabrese et al. 2016). To reduce the bias from irregular time intervals, optimally weighted AKDEs (i.e., oversampled times were down‐weighted and undersampled times were up‐weighted) were estimated and the 95% utilization distribution (UD) isopleth (i.e., contour line) was retained as the estimate of segment‐level space use (Fleming et al. 2018). The 95% UD is used in assessments of animal spatial ecology to represent the region containing 95% of the total density estimated by the selected method. Because we are estimating the home range using AKDE, this estimate represents the area traversed by an animal during its normal activities (Burt 1943; Fleming et al. 2015; Hooten et al. 2017). An error model was not implemented given the low level of spatial location error for these data. Additionally, the best‐fitting CTMMs were used to estimate regularized (i.e., all time intervals equal) positions at a 30‐min time interval to increase the temporal resolution of the available data and fill any large gaps (Yang et al. 2023).

Population‐level space use was estimated on a seasonal basis to evaluate phenological patterns of potential overlap with poultry farms by all tagged mallards moving through the Mississippi Flyway. This was conducted using the segment‐level estimates of ‘local movements’ as input via the *ctmm* R package (Calabrese et al. 2016), which represented periods of movements where mallards were more likely to come into contact with farms or the surrounding area. Seasons of the full annual cycle (breeding, post‐breeding migration, non‐breeding, pre‐breeding migration) for mallards were defined using an *ad hoc* approach, and profiles of mallard displacement over time were inspected in comparison to dates delimiting seasons from other sources (Fink et al. 2022; Roberts et al. 2023; Teitelbaum et al. 2023). Based on this process, breeding/pre‐migration (May 1–October 31), fall migration (November 1–November 30), wintering (December 1–February 28), and spring migration (March 1–April 30) were defined to generally match the phenology of tracked mallards. Population‐level space use was estimated by calculating the average aggregate UD across all track segments that fell within a particular season using the *mean.UD()* function from the *ctmm* package (Calabrese et al. 2016); segment‐level UDs that fell across multiple seasons were attributed to the season that represented the majority of the track segment.

### 2.4 Spatiotemporal Overlap of Mallards With Domestic Poultry Farms

Using both regularized mallard tracks (i.e., points) and space use estimates (i.e., 95% UDs) from ‘local movements’, overlap with poultry farms was evaluated over space and time for each individual mallard; hereafter referred to as observation‐level overlap (for points) and segment‐level overlap (for UDs). Locations of poultry farms with confirmed infection of HPAI H5N1 attributed to independent wild bird introduction (farms that genetically indicated potential for farm‐to‐farm transmissions were excluded) during 2022 were obtained from the USDA National Veterinary Services Laboratories (NVSL) for the conterminous U.S. This dataset included NVSL‐identified wild bird spillover events of HPAI H5N1 at both commercial and backyard poultry farms (Youk et al. 2023). Observation‐ and segment‐level overlap was assessed using a sensitivity analysis to account for uncertainty in farm size, pathogen movement, and the duration of virus viability in the environment. This was performed by setting a buffer around the coordinates of each farm location that varied in size (1‐, 3‐, 5‐km radius), as well as defining an exposure period (14, 30, 90 days) prior to confirmation of HPAI H5N1 detection at a poultry farm. Given the varying dimensions of farms where poultry or farm workers may have access, it was assumed that shorter distances (< 1 km) were more likely to be associated with a spillover event than longer distances (~5 km). Likewise, prior studies on the environmental persistence of AIV found that virus isolates can remain infectious for months (Keeler et al. 2014; Poulson et al. 2024; Ramey et al. 2020), especially in cold freshwater wetlands that serve as breeding habitat for mallards within the Mississippi Flyway. We also explored how overlap would change over three exposure periods of increasing duration: the expected infectious period of 14 days in mallards (Spackman et al. 2023), a duration slightly longer than the infection period where the virus was likely to remain infectious (30 days), and the upper duration of environmental persistence (~90 days) measured by Keeler et al. (2014). However, this 90‐day period is still relatively conservative compared to other studies showing that the virus can persist across seasons (Poulson et al. 2024; Ramey et al. 2020). For measures of observation‐level overlap, positions that fell within the spatiotemporal bounds of the farm buffer and exposure period (e.g., within 1 km and 14 days of a reported spillover event) were considered to overlap a given farm. When evaluating segment‐level overlap, the simultaneous intersection of the 95% UD with the farm buffer and the duration of the track segment with the exposure period was considered overlapping in space and time.

We also evaluated seasonal overlap of mallard population‐level space use with poultry farms (regardless of HPAI spillover event status) to better understand when and where mallards within the Mississippi Flyway may be likely to overlap with these farms. This is expected to provide predictions on a seasonal basis of farms that may be at greatest risk of HPAI spillover events. To perform this analysis, we measured overlap between population‐level mallard UDs and locations of poultry farms predicted by the USDA hybrid model (Patyk et al. 2020). As with the NVSL dataset, the USDA hybrid model dataset included a variety of backyard and commercial poultry farm categories with locations that were predicted to share landscape characteristics with true poultry farms (Patyk et al. 2020). Similar to the methods applied for quantifying observation‐ and segment‐level overlap, we conducted a sensitivity analysis by varying buffer size around the farm locations (1, 3, and 5 km). A sensitivity analysis on exposure period was not needed, given that the overlap with poultry farms was only spatial in nature after estimating mallard population‐level space use by season.

### 2.5 Estimation of HPAI Spillover Probability Into Poultry Farms

We investigated the probability of an HPAI H5N1 wild bird spillover event occurring as a function of farm type and mallard overlap, while generally accounting for steps that may preclude the final reporting of a spillover event (i.e., detection), within an occupancy model framework (MacKenzie et al. 2002). This approach can enable a better understanding of the potential effect of these tagged mallards (and other untagged waterfowl that use the same resources) on previously reported spillover of HPAI. Additionally, it also provides insight as to which types of poultry farms (e.g., backyard, commercial, turkey, broiler, layer) may be at greatest risk of experiencing HPAI spillover from wild birds. For a spillover event to ultimately be reported by USDA, domestic poultry must first exhibit clinical signs of infection, which would need to be observed and reported by farm operators to animal health officials. Then, molecular testing by the National Animal Health Laboratory Network (NAHLN), with confirmation by NVSL, would lead to an official report of a spillover event (Pepin et al. 2014). Because the outcomes of these individual steps are unknown, we represent this entire reporting pathway using a single variable (hereafter referred to as the ‘probability of detection’) and estimate this parameter in the model for backyard farms only because they are much more likely not to report events to government officials (McClaughlin et al. 2024; Souvestre et al. 2021). This analysis was fit using a Bayesian hierarchical model such that:

$$\begin{array}{l} \text{logit}\left(p_{i}\right)=\beta_{0}\\ \text{logit}\left(\psi_{j}\right)=\alpha_{0}+{\text{Overlap}}_{j}\alpha_{1}+{\text{FarmType}}_{\mathrm{jk}}\tau_{\mathrm{jk}}\\ z_{i}∼\text{Bernoulli}\left(p_{i}\right)\\ y_{j}∼\text{Bernoulli}\left(z_{j}\times \psi_{j}\right), \end{array}$$

where $p_{i}$ is the probability of detection for an HPAI event at backyard farm i; all commercial farms were assumed to have a probability of detection of 100% given the high levels of morbidity and mortality among domestic poultry and the degree of active and passive surveillance conducted in 2022 during the HPAI H5N1 U.S. outbreak. The probability of detection model for backyard farms used a simple intercept‐only function ($\beta_{0}$) given the limited data available, which would reflect differences in reporting for this farm type that was available across both the NVSL and USDA hybrid model datasets. Spillover probability ($\psi_{j}$) was estimated as a function of mallard overlap with poultry farms (i.e., overlapping or non‐overlapping) and farm type (i.e., backyard or commercial farm) for each farm j, where $\alpha_{0}$ represents the intercept, $\alpha_{1}$ represents the coefficient for the ‘overlap’ predictor variable, and a random effect ($\tau_{\mathrm{jk}}$) was included for commercial farm subtype k (i.e., turkey, broiler, layer). The latent response variable $z_{i}$ represents whether a spillover event was detected or not based on the probability of detection $p_{i}$, whereas the response variable $y_{j}$ denotes whether an HPAI event was confirmed or not (i.e., observed) for a given farm as a product of the latent state z (0 = no spillover detected; 1 = spillover detected) and the spillover probability ψ (continuous value between 0 and 1, where values near zero represent very small probability of spillover whereas values near 1 indicate very high probability of spillover occurring).

For this model, we used a combination of the locations of confirmed HPAI H5N1 detections in poultry attributed to wild bird introductions from the NVSL dataset (y=1) and the USDA hybrid model locations (y=0) to estimate the probability of H5N1 spillover. The USDA hybrid model dataset is reflective of all potential poultry farms throughout the conterminous U.S., which would include locations reported in the NVSL dataset. Therefore, we removed any USDA hybrid model location that fell within 5 km of a data point in the NVSL dataset due to the likelihood that they represented the same farm. Because the geographic extent of the tracked mallard movements (i.e., Mississippi Flyway) was limited, only farms that were within states overlapped by mallard tracks were retained for further analysis. This was performed to potentially remove any other spatial effects that were not accounted for by the model. After this filtering process, we assessed segment‐level mallard overlap (0 = no overlap; 1 = overlap) with both sets of poultry farm locations (NVSL, USDA hybrid model) using a 5‐km buffer radius around farms. Backyard farms were analyzed as a single group (i.e., not divided by species or subtype) from both the NVSL outbreak and USDA hybrid model datasets, whereas commercial farms were separated further into poultry farm subtypes (i.e., turkey, layer, broiler).

The hierarchical occupancy model was fit with the *rstan* R package (Stan Development Team 2023), where the no‐U‐turn sampler (NUTS) (Hoffman and Gelman 2014) from Hamiltonian Monte Carlo was used via Stan. The latent variable z was marginalized out of the model because Stan cannot generate a gradient to estimate values for discrete states (Monnahan et al. 2017; Yackulic et al. 2020). Weakly informative priors (on the link scale) were used to improve parameter estimation (Lemoine 2019; McElreath 2020), which was supported by prior predictive simulations and successfully recovering true parameter values from simulated data (Gabry et al. 2019; Wesner and Pomeranz 2021). We ran four Markov chains using a total of 4000 iterations, where the first 2000 were treated as the warmup, resulting in 8000 samples from the posterior distribution. Model convergence was assessed by inspecting trace plots of the parameters, effective sample size, and $\hat{R}$ (where $\hat{R}$ < 1.01 is indicative of model convergence by multiple chains exhibiting stationarity and reaching a common distribution), whereas posterior predictive checks were performed to assess goodness‐of‐fit (Gabry et al. 2019; Vehtari et al. 2021). Estimated parameters were considered statistically significant if the 95% Bayesian credible intervals did not overlap zero. After the model was run, trace plots exhibited stationarity, effective sample size was relatively high, and all $\hat{R}$ values were ≤ 1.002, indicating the model had converged on the posterior distribution. Additionally, posterior predictive checks showed that model predictions were in strong agreement with the data, which implied that the model was properly specified.

## 3 Results

### 3.1 Observation‐Level Overlap

**FIGURE 3:**
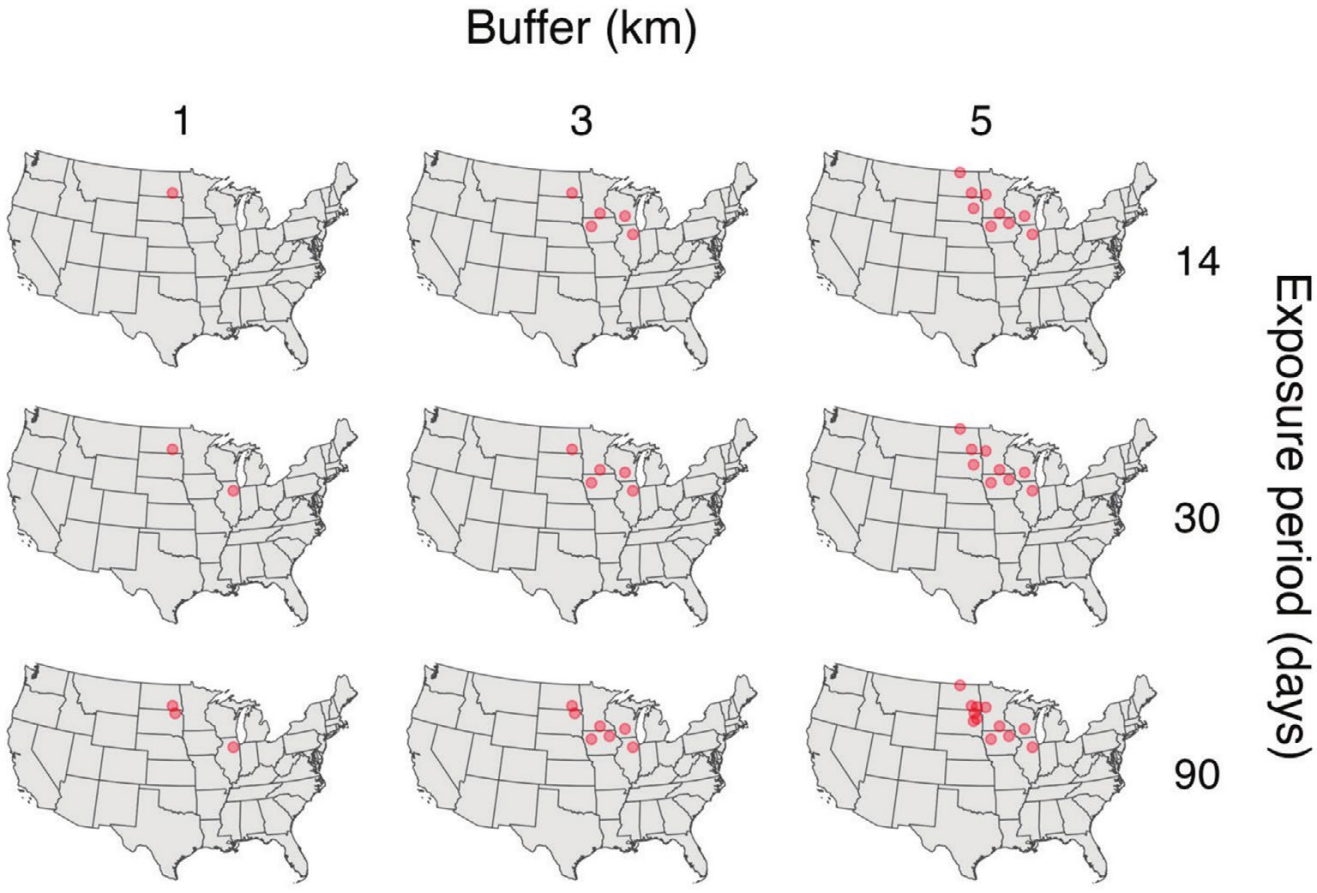
Locations of HPAI H5N1 spillover events at poultry farms that overlapped with individual mallard observations. Overlap was assessed via a sensitivity analysis that varied buffer size around farms and exposure period before a spillover event was reported, resulting in nine different measures of overlap. The basemap was created using data from Natural Earth.

**FIGURE 4:**
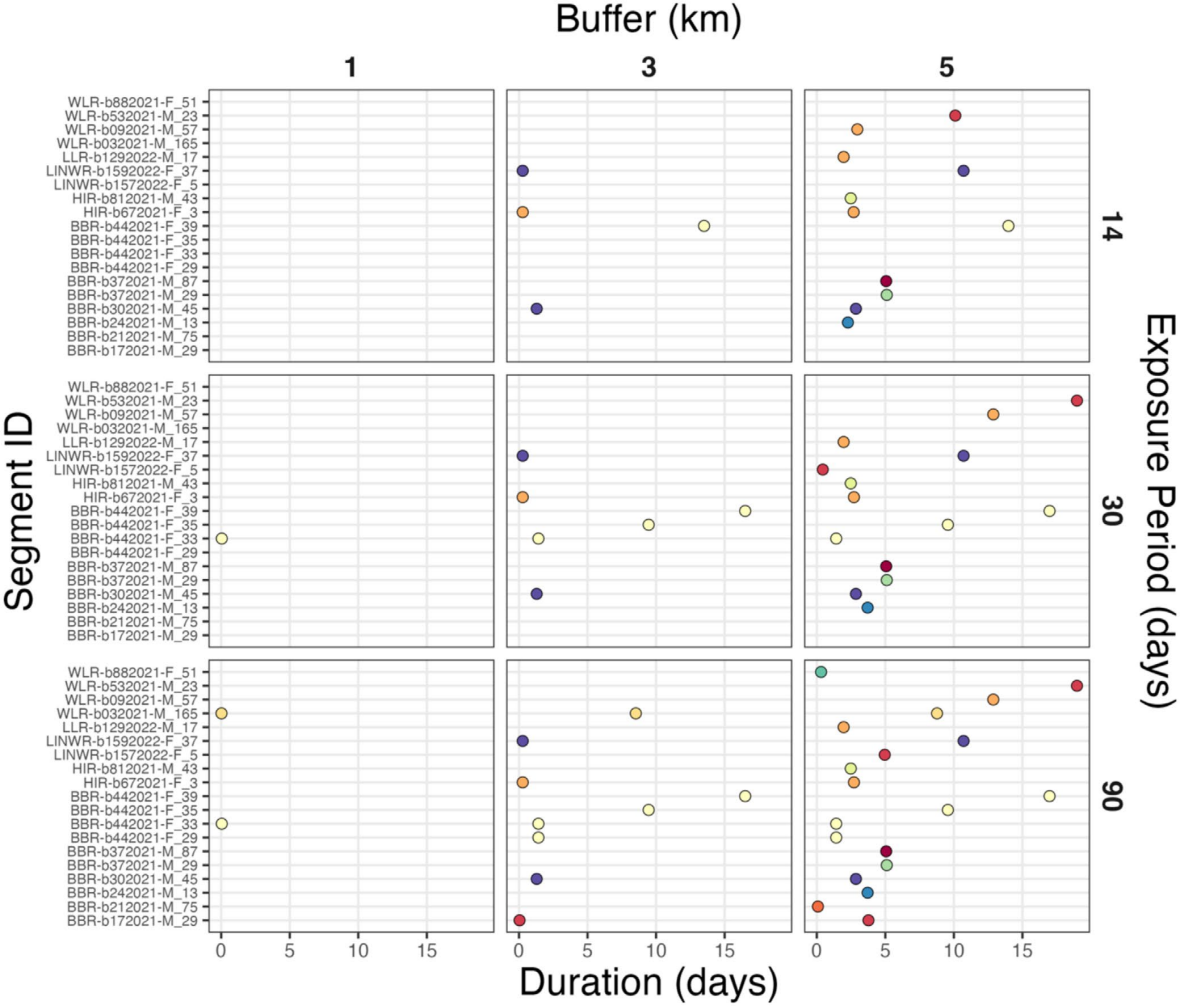
Overlap duration of mallard observations (by track segment ID) with poultry farms (denoted by different colors for points) that experienced HPAI H5N1 spillover events in 2022 is shown following the sensitivity analysis (combinations of three buffer distances and three exposure periods). Track segments that consisted only of a single overlapping observation are not shown because duration could not be calculated for a single point. Some individual birds are included twice on the *y*‐axis, but for different segments over the entire track length (where the number on the end denotes the segment number and all previous text represents the mallard ID).

Based on the sensitivity analysis, buffer size appeared to be more influential than exposure period in explaining the number of farms overlapped. At the most conservative end of the sensitivity analysis (1‐km buffer, 14‐day exposure period), a single observation from one mallard track overlapped with one farm. By comparison, 15 mallard tracks overlapped with 12 farms when using a buffer of 5 km and an exposure period of 90 days (Figure 3). Of these overlapping tracks, nine exhibited an overlap duration of ≥ 5 days when using a 5‐km buffer and 90‐day exposure period (Figure 4). Alternatively, only two farms were overlapped for ≤ 1.5 h when using a 1‐km buffer with an exposure period of 90 days (Figure 4). In some cases, mallards only overlapped with poultry farms by a single observation (likely indicative of no relevant overlap), whereas other individuals overlapped for up to 19 days at a time with over 800 overlapping observations (at a 30‐min time interval) (Figure S1; Table S1). Although we expect that birds at the shortest buffer distances and exposure periods (e.g., 1 km, 14 days) would be associated with a greater probability of a spillover event, we cannot rule out the potential role of other mallards or untagged waterfowl as potential sources of HPAI exposure. Additionally, the majority of observation‐level overlap with poultry farms occurred during the spring migration season (58%), followed by three instances of overlap during the breeding season (25%), and only two instances of overlap occurring during the fall migratory season (17%). All overlapped farms were found in the Midwest and Prairie Pothole Region of the United States (Figure 3).

### 3.2 Segment‐Level Overlap

**FIGURE 5:**
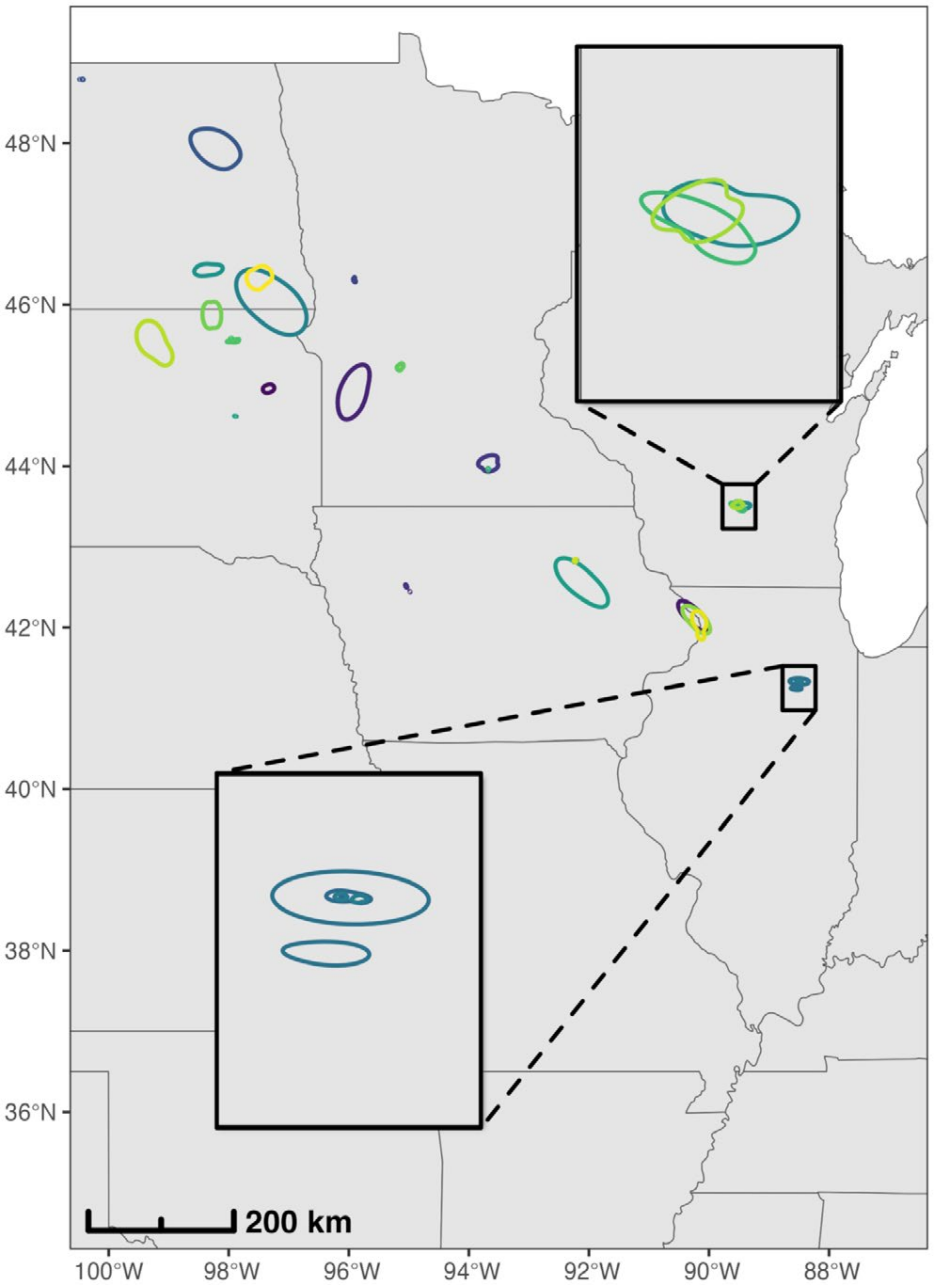
Map of segment‐level mallard 95% utilization distributions (UDs) that overlapped with poultry farms that were positive for HPAI H5N1 in 2022 using a 5‐km buffer and 90‐day exposure period. Due to the sensitive nature of these data, farm locations are not shown. Contours denote segment‐level mallard UDs by mallard ID (colors) and represent the home range of mallard movement for that particular track segment. Some individual birds have multiple UDs overlapping farms that experienced spillover events. Magnified regions show overlap among multiple birds for a single farm in Wisconsin (top) and separate visitations from a single bird near a farm in Illinois (bottom). The basemap was created using data from Natural Earth.

Segment‐level overlap of mallards with poultry farms showed a similar result as the observation‐level analysis, where only a limited number of farms experienced spatiotemporal overlap by mallards and buffer size was more influential than exposure period for the sensitivity analysis. When using a 1‐km buffer and 14‐day exposure period, 11 mallard UDs overlapped with 10 poultry farms (Figure S2). At the other end of the spectrum, 30 mallard UDs overlapped with 24 farms when using a 5‐km buffer and an exposure period of 90 days. As was found for the observation‐level analysis, increases in buffer size led to a greater number of overlapped poultry farms compared to increases in exposure period. All overlapped farms were located in the Midwest and Prairie Pothole Region (Figure S2). In some instances, multiple mallards overlapped with a single farm for a given spatiotemporal window, or a single mallard returned to the same farm location during non‐consecutive periods of range residency (i.e., local movements; Figure 5).

### 3.3 Seasonal Population‐Level Overlap

**FIGURE 6:**
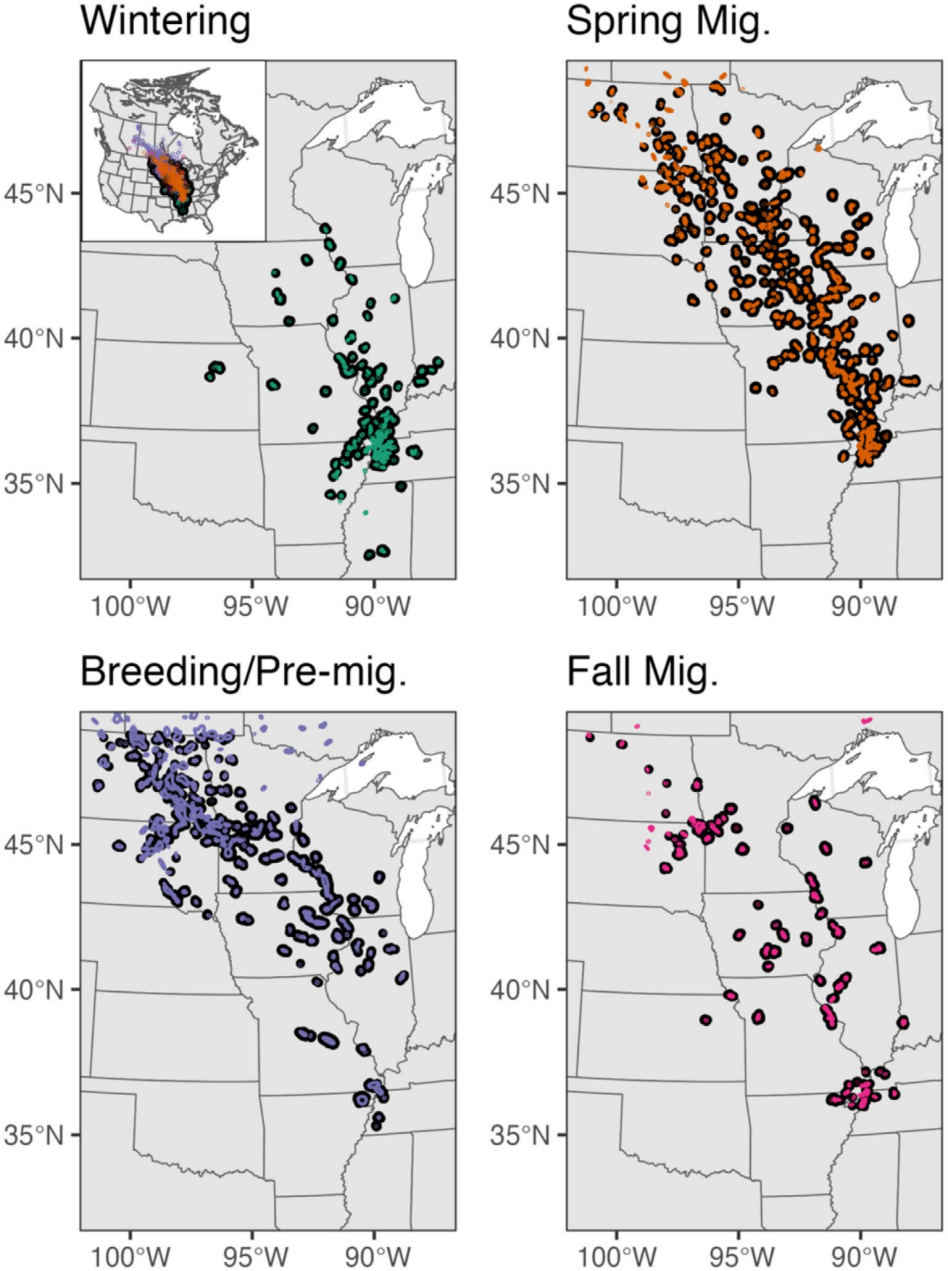
Population‐level utilization distributions (UDs) of local movements by mallards (colored contours) are shown with respect to overlapped poultry farms as estimated by the USDA hybrid model (black points) on a seasonal basis. These seasonal UDs collectively represent areas of localized mallard movement and serve as a sample of the wider population. In this example, overlap was assessed using a 5‐km buffer around farms, where mapped farms denote the point location rather than the buffer itself to improve visualization of overlap by mallards. Seasons are defined as breeding/pre‐migration (May 1—October 31), fall migration (November 1—November 30), wintering (December 1—February 28), and spring migration (March 1—April 30). Inset map shows full extent of mallard movements. The basemap was created using data from Natural Earth.

We detected differences across seasons in the number of estimated poultry farms (derived from the USDA hybrid model) that overlapped by mallard population‐level space use. Of the 6431 overlapped farms (using a 5‐km buffer), 44% occurred during the spring migratory season and 32% during the breeding/pre‐migration season (Figures 6 and S3). By comparison, the winter (13%) and fall migratory (11%) seasons represented a much smaller proportion of overlapped farms. Additionally, 185 of the 6431 overlapped farms (2.9%) were those with a confirmed HPAI H5N1 spillover event during our study period when using a 5‐km buffer, whereas only 54 farms (< 1.0%) with a confirmed spillover event were overlapped when using a 1‐km buffer. The geographic distribution of overlapped farms varied predictably across seasons, where mallards were concentrated in the Lower Mississippi Alluvial Valley during the winter season and within the Prairie Pothole Region during the breeding/pre‐migration season (Figures 6 and S3).

### 3.4 HPAI Spillover Probability at Poultry Farms

**FIGURE 7:**
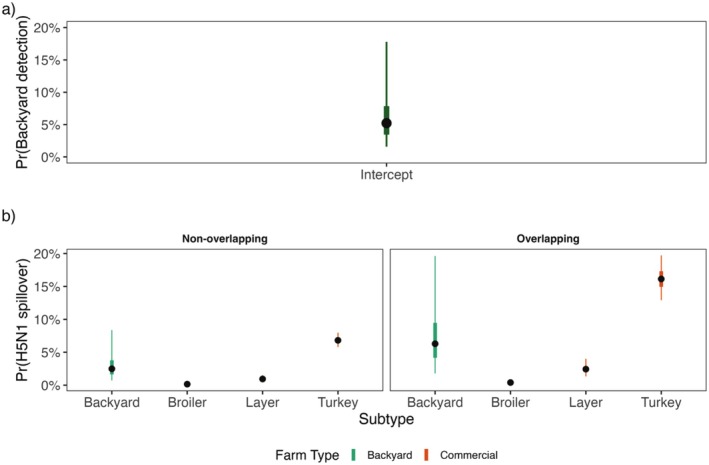
Results of the Bayesian hierarchical model estimating probability of HPAI H5N1 spillover events at poultry farms accounting for rates of reporting HPAI spillover events. (a) Estimated HPAI probability of detection for backyard farms based on a simple intercept‐only model. (b) Estimated HPAI H5N1 spillover probability based on farm type and whether farms were (or were not) overlapped by mallard space use. Black points denote the median effect, and the colored bars denote the 50% and 95% credible intervals.

The Bayesian hierarchical model estimated the probability of detection (encompassing steps from detection of clinical signs through testing and confirmation by NVSL) for HPAI in backyard poultry operations, as well as differences in spillover event probability among farms. The model estimated that the median HPAI probability of detection for backyard poultry farms was quite low at 5.2% (95% CI: 1.5%–18.6%) (Figure 7a). Moreover, farms that were overlapped by mallard segment‐level space use were associated with up to a 2.6‐fold (95% CI: 2.0–3.4) higher probability of H5N1 spillover compared to farms not overlapped by mallards. Regardless of overlap status with mallards, backyard farms had a greater median probability of H5N1 spillover (overlap: 6.3%; no overlap: 2.5%) compared to commercial broiler (overlap: 0.4%; no overlap: 0.2%) and layer operations (overlap: 2.4%; no overlap: 0.9%); however, commercial turkey farms were associated with the highest spillover probability overall (overlap: 16.1%; no overlap: 6.8%; Figure 7b). It is worth noting that the probability estimate for backyard farms was associated with a large degree of uncertainty, likely related to the use of an intercept‐only probability of detection parameter that did not use any covariates to inform estimation of reporting probability for this farm type.

## 4 Discussion

When evaluating the probability of HPAI H5N1 spillover at poultry farms, spatial overlap by mallards significantly increased the likelihood of transmission risk. Farms overlapped by mallards were up to 2.6 times (95% CI: 2.0–3.4) more likely to have a confirmed HPAI H5N1 spillover event compared to those that were not overlapped; but it should be noted that these estimates are for the first year of the current outbreak, and prevalence of HPAI in wild waterfowl is expected to change over time (Harvey et al. 2023; Ramey et al. 2018). The risk estimates reported in this study do not imply a causal relationship of tagged mallards representing the source of recorded spillover events. Importantly, these tracked mallards are a proxy for untagged mallards and other wild waterfowl that were infected with HPAI and represented the ultimate source of the spillover at poultry farms. This relationship suggests that mallards may also be an indicator species for waterfowl aggregation sites more generally, which could be used to identify high‐risk areas for HPAI spillover events. In particular, indirect transmission may be a major source of infection, as has been found in previous studies (Silk et al. 2018; Viana et al. 2014; Wibawa et al. 2018). Some of these modes of indirect transmission include humans coming into contact with landscape features (i.e., ponds, wetlands, fields) that are contaminated by infected waterfowl and then exposing poultry through different possible routes, poultry coming into contact with fecal matter from wild birds that had previously visited the land surrounding the farm, as well as other potential routes of exposure (Guinat et al. 2020; Mateus‐Anzola et al. 2023; Wibawa et al. 2018).

Commercial turkey farms and backyard poultry farms were estimated to be at the greatest risk of HPAI H5N1 infection compared to commercial layer and broiler operations. Besides the geographic distribution of commercial turkey farms being relatively high in the Midwest and Prairie Pothole Region, where mallards tend to migrate and breed within the Mississippi Flyway (USDA NASS 2023), turkey flocks may be particularly prone to AIV due to their high viral transmission and shedding rates (Kirkeby et al. 2024; Pillai et al. 2010). Additionally, previous studies on experimental infection and the underlying genetic vulnerability of poultry found that the virulence of AIV was higher in turkeys than in chickens (Blaurock et al. 2022; Pantin‐Jackwood et al. 2017). Therefore, our results support the higher probability of HPAI spillover events occurring in commercial turkey flocks compared to commercial chicken farms. Backyard farms are also expected to be at greater risk of HPAI spillover events because poultry with outdoor access have an increased likelihood of exposure to infected wild birds and viable virus in the environment (Pepin et al. 2014). Given these findings, evaluating the efficacy of specific farm‐level biosecurity measures and wild bird mitigation strategies (e.g., reducing pooled water, use of dogs as a deterrent, minimizing wild bird attractants) could help reduce the risk of HPAI spillover at commercial and backyard poultry farms.

This study provides an initial estimate for the probability of detection (i.e., the product of all steps from observation of clinical symptoms through testing by NVSL) of HPAI H5N1 in backyard poultry farms, which was estimated to be quite low (median: 5.2%; 95% CI: 1.5%–18.6%) compared to commercial operations. This low probability and high uncertainty could be due to numerous reasons, such as flock type and size, as well as unfamiliarity with clinical signs of avian influenza or legal requirements related to compliance with biosecurity measures by backyard farm operators (McClaughlin et al. 2024; Souvestre et al. 2021). Additionally, high uncertainty in this estimate was also related to the use of an intercept‐only linear model due to the lack of relevant covariates available for the full dataset. However, it is worth noting that the contribution of backyard poultry farms to large‐scale outbreaks of AIV is relatively low in comparison to commercial operations (Bavinck et al. 2009; Souvestre et al. 2019; Thomas et al. 2005), so the focus on reduction of spillover events at commercial poultry farms is expected to have a greater overall control on stemming the progression of an AIV outbreak. Given the high risk of backyard farms to HPAI spillover events (Pepin et al. 2014), understanding the reasons for underreporting of HPAI from backyard farms could be investigated. This may include surveys of farmers to evaluate their knowledge of AIV and associated clinical signs of infection in poultry or their willingness to report suspected infections to government agencies.

At the population level, tracked mallards exhibited spatial overlap with many poultry farms identified in the USDA hybrid model and located throughout the Mississippi Flyway. Despite the limited number of farms with spillover events that were overlapped by tagged mallards, there is the potential for more frequent indirect interactions between domestic poultry and untagged mallards (or other wild waterfowl) that also use these habitats and migratory flyways. Although this study focused on a single species and flyway, similar patterns of overlap and spillover risk might be observed within mallards in other flyways and possibly some other dabbling ducks (Anatidae) because they are attracted to similar types of wetland habitat and act as natural reservoirs of AIV (Kent et al. 2022; McDuie et al. 2022, 2024; Prosser et al. 2024), though such relationships would need to be validated with future work. Overlap was likely during the spring migratory and breeding/pre‐migration seasons, which comprised ~75% of all farms that were overlapped by population‐level space use by mallards. Of relevance, mallards often settled at breeding sites in the upper Midwest and Prairie Pothole Region of the United States and Canada, which overlapped with previously identified hotspots of high AIV prevalence based on wild bird surveillance between 2007 and 2019 (Bevins et al. 2014; Kent et al. 2022). By comparison, the space use of mallards during the winter season was low and constrained to the Lower Mississippi Alluvial Valley, where AIV prevalence has been predicted to peak during the winter and early spring when mallards occupy this region (Bevins et al. 2014; Kent et al. 2022). The consistent spatiotemporal trend of AIV prevalence in wild waterfowl across years supports the notion that the dynamics of AIV geographic spread can be very similar over time regardless of the particular outbreak, at least for endemic viral strains. This relationship could be leveraged to inform AIV surveillance strategies and to dynamically heighten biosecurity measures at poultry farms that are expected to be at greatest risk of spillover events over time and space.

When focusing on mallard relocations (i.e., observation‐level overlap), there were few instances of extended time periods of overlap with poultry farms—the longest instance lasting upwards of 19 days (5‐km buffer, 90‐day exposure period) (Figure S1; Table S1). These patterns were similar to those using the UDs of mallard range residency periods (i.e., segment‐level overlap), which showed a slightly greater number of farms overlapped due to the use of relatively larger polygons as opposed to individual relocations. Despite several instances of extended spatiotemporal overlap of tagged mallards with poultry farms, the vast majority (94%) of NVSL‐identified wild bird spillover events of HPAI H5N1 were likely the result of other sources. This highlights the role of the tagged mallards serving as a proxy for waterfowl movement patterns and evaluating the potential HPAI spillover risk they impose.

Although this study provides new evidence in the assessment of spatiotemporal overlap of wild birds in relation to confirmed wild bird spillover events of HPAI H5N1 into poultry farms, previous studies have pursued similar objectives across taxonomic groups and different geographies. For example, McDuie et al. (2022) examined movements of 11 waterfowl species and found overlap with commercial livestock facilities, wherein several tracked birds used livestock facilities (e.g., stock tanks), nearby retention ponds, and in some cases exhibited high annual site fidelity. Likewise, wild blue‐winged teal (*Spatula discors*) were found to overlap with poultry farms seasonally (Humphreys et al. 2021). Analyzing wild bird movements across larger geographic extents and taxonomic breadth, instead of isolated studies, could support identification of species that contribute most to potential spillover of HPAI into poultry farms. This type of analysis could provide a more comprehensive perspective on HPAI spillover risk due to wild bird introductions, which could be used to inform more targeted biosecurity measures at poultry farms. Additional fine‐scale work that evaluates specific routes of HPAI exposure on poultry premises could lead to more targeted interventions.

Although our findings suggest mallard overlap with poultry farms is relatively extensive throughout the Mississippi Flyway and overlap is associated with an increased risk of HPAI outbreaks, we relied on several assumptions during our analysis. Despite using a sensitivity analysis to account for varying values of buffer distance around farms or exposure periods preceding confirmed poultry detections, it is possible that these values are not reflective of exposure to HPAI at some of these farm locations. Additionally, the population‐level estimates of seasonal space use by mallards assumed that they exhibit fidelity to sites (i.e., breeding, wintering, stopover) and migratory flyways (Roberts et al. 2023); otherwise, these estimates would not be predictive outside of the study period. Lastly, our spillover risk model assumes the HPAI probability of detection is the same at all backyard poultry farms. Although this may be the case for commercial farms due to regulations and increased biosecurity measures, there is likely greater heterogeneity in reporting among backyard poultry producers.

This study provides evidence further supporting the integration of movement ecology and disease ecology to better understand the spread and transmission of disease. We demonstrate the potential for increased risk of HPAI spillover events at poultry farms related to spatiotemporal overlap with wild waterfowl. Given the increase of natural and anthropogenic disturbances that drive the movement of wild animals, often into close contact with humans, interdisciplinary studies can support better prediction of disease dynamics and methods by which risk may be reduced.

## Author Contributions

**Joshua A. Cullen:** conceptualization (equal), formal analysis (lead), investigation (equal), methodology (lead), software (lead), visualization (lead), writing – original draft (lead), writing – review and editing (equal). **Nicholas M. Masto:** conceptualization (equal), data curation (equal), methodology (supporting), writing – review and editing (equal). **Jeffery D. Sullivan:** conceptualization (equal), investigation (equal), methodology (supporting), software (supporting), writing – review and editing (equal). **Cory J. Highway:** data curation (equal), writing – review and editing (equal). **Kelly A. Patyk:** data curation (equal), writing – review and editing (equal). **Mary‐Jane McCool:** data curation (equal), writing – review and editing (equal). **Mia Kim Torchetti:** methodology (supporting), writing – review and editing (equal). **Kristina Lantz:** methodology (supporting), writing – review and editing (equal). **Rebecca L. Poulson:** methodology (supporting), writing – review and editing (equal). **Deborah L. Carter:** methodology (supporting), writing – review and editing (equal). **Jamie C. Feddersen:** data curation (equal), writing – review and editing (equal). **Bradley S. Cohen:** data curation (equal), funding acquisition (lead), writing – review and editing (equal). **Diann J. Prosser:** conceptualization (equal), investigation (equal), writing – review and editing (equal).

## Conflicts of Interest

The authors declare no conflicts of interest.

## Supporting information


**Appendix S1:** ece372221‐sup‐0001‐AppendixS1.docx.

## Data Availability

Data supporting this manuscript are not released publicly to protect the privacy of individual producers that could be identified through this information. Contact the corresponding author (Diann Prosser, dprosser@usgs.gov) for more information and to be directed to appropriate contacts for individual datasets based upon your specific needs.
