# Supplementary material for: Spatiotemporal Overlap of Mallards With Poultry Farms Is Associated With Greater Risk of Avian Influenza Wild Bird Spillover Events
Source: Ecol Evol. 2025 Oct 1;15(10):e72221. doi: 10.1002/ece3.72221 (PMC12486340; doi:10.1002/ece3.72221)
Supplement: Supplementary file 1 — Appendix S1: ece372221‐sup‐0001‐AppendixS1.docx. [file ECE3-15-e72221-s001.docx]

Supporting Information

**Spatiotemporal overlap of mallards with poultry farms is associated with greater risk of avian influenza wild bird spillover events**

Joshua A Cullen^1^, Nicholas M Masto^2^, Jeffery D Sullivan^3^, Cory J Highway^2^, Kelly A Patyk^4^, Mary-Jane McCool^4^, Mia Kim Torchetti^5^, Kristina Lantz^5^, Rebecca L Poulson^6^, Deborah L Carter^6^, Jamie C Feddersen^7^, Bradley S Cohen^2^, Diann J Prosser^3^

^1^ORISE Fellow at U.S. Geological Survey, Eastern Ecological Science Center, Laurel, MD, USA

^2^College of Arts and Sciences, Tennessee Technological University, Cookeville, TN, USA

^3^U.S. Geological Survey, Eastern Ecological Science Center, Laurel, MD, USA

^4^U.S. Department of Agriculture, Animal and Plant Health Inspection Service, Veterinary Services, Strategy and Policy, Center for Epidemiology and Animal Health, Fort Collins, CO, USA

^5^U.S. Department of Agriculture, Animal and Plant Health Inspection Service, National Centers for Animal Health, National Veterinary Services Laboratories, Ames, IA, USA

^6^Southeastern Cooperative Wildlife Disease Study, College of Veterinary Medicine, University of Georgia, Athens, GA, USA

^7^Tennessee Wildlife Resources Agency, Nashville, TN, USA

Ecology and Evolution


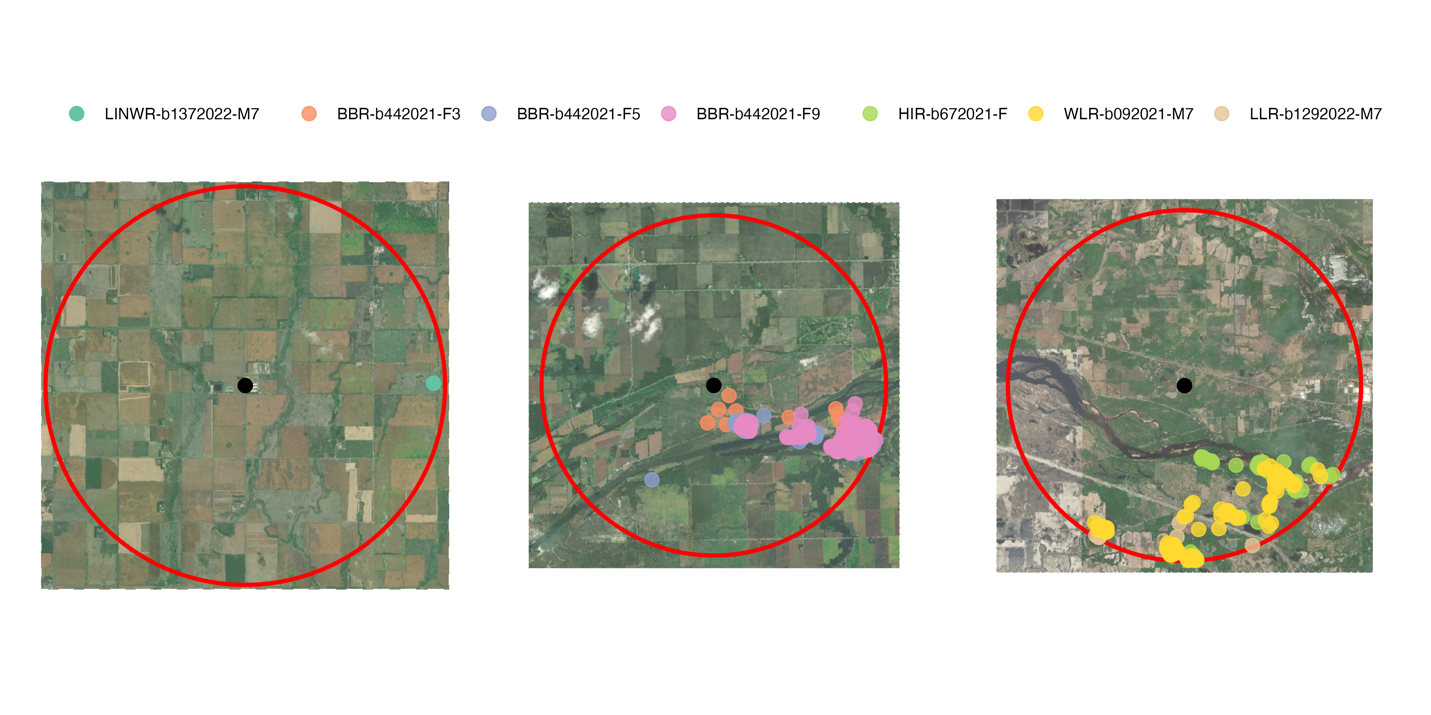


**Figure S1.** Detailed views of mallard overlap with three poultry farms that reported spillover events of HPAI during the study period (2022). Black points denote the farm location, whereas red circles represent a 5-km buffer around each farm. Colored points denote overlapping locations of estimated mallard movements (at a 30-min time interval) based on this 5-km buffer and a 90-day exposure period. From left to right, farms are labelled as Farm A, Farm B, and Farm C. The basemap was created using data from Esri World Imagery (https://services.arcgisonline.com/ArcGIS/rest/services/World_Imagery/MapServer).

**Table S1.** Summary of overlap between subset of mallard locations (at 30-min interval) and poultry farms that reported HPAI spillover events during the study period (shown in Figure S1). Overlap was assessed using a 5-km spatial buffer and 90-day prior exposure period. Spillover period is reported (year-month) instead of date due to sensitive nature of data.

| Farm ID | Farm Type | Spillover Period | Number of overlapping mallards | Number of overlapping relocations |
| --- | --- | --- | --- | --- |
| Farm A | Commercial | 2022-04 | 1 | 1 |
| Farm B | Backyard | 2022-11 | 1 | 1,339 |
| Farm C | Backyard | 2022-04 | 3 | 689 |


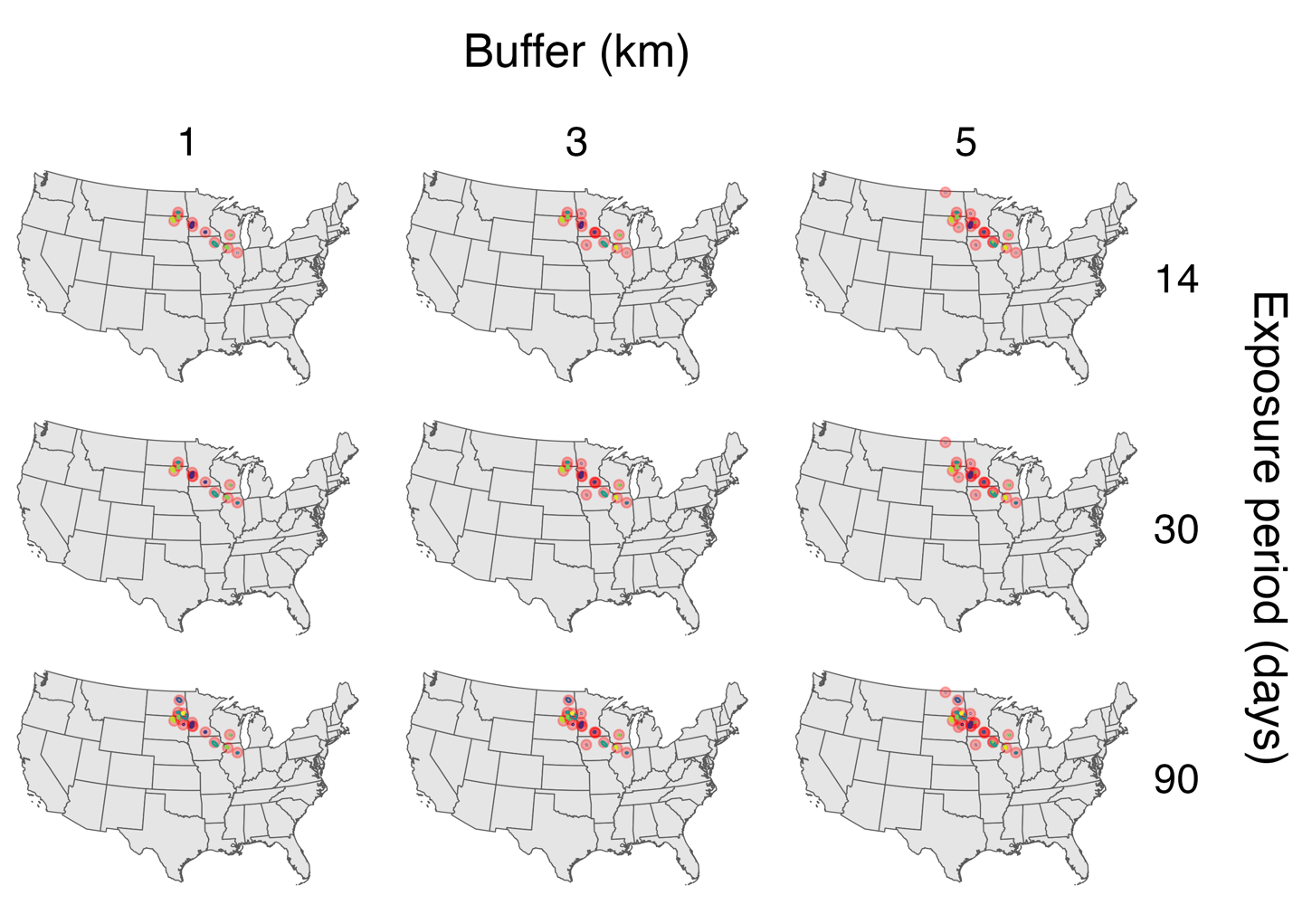


**Figure S2.** Locations of HPAI H5N1 spillover events at poultry farms that overlapped with segment-level ranges of mallards. Overlap was assessed via a sensitivity analysis that varied buffer size around farms and exposure period before an outbreak was reported. Red points denote overlapped farms, whereas colored contours denote segment-level home ranges by mallard ID. Number of overlapping UDs ranged from 11 to 30, whereas the number of overlapped farms with spillover events ranged from 10 to 24. The basemap was created using data from Natural Earth.


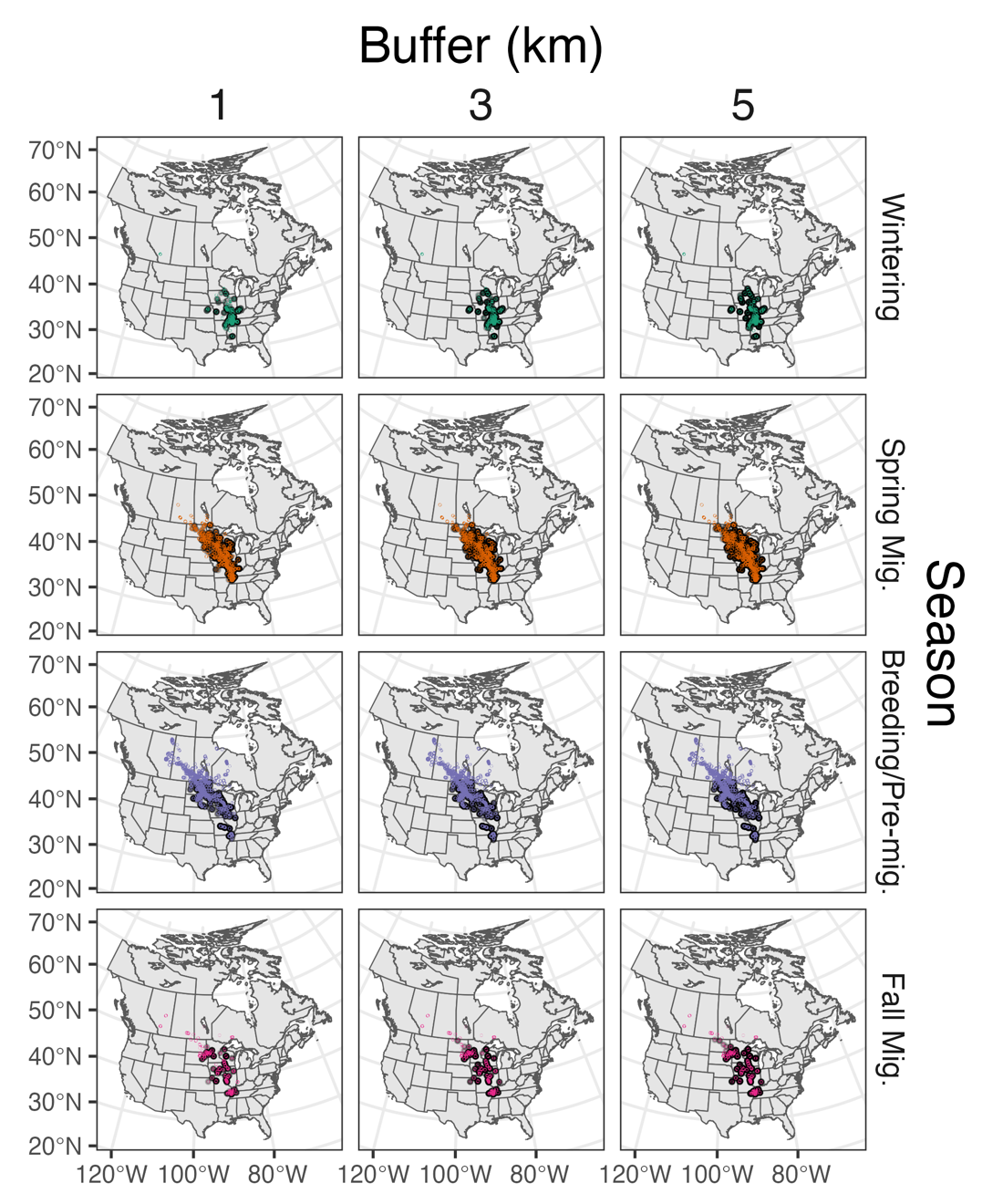


**Figure S3.** Population-level space use of mallards by season of the full annual cycle with respect to overlapped poultry farms (predicted by USDA hybrid model). Colored contours represent mallard space use, whereas black points denote overlapped poultry farms. Seasons are defined as breeding/pre-migration (May 1 – October 31), fall migration (November 1 – November 30), wintering (December 1 – February 28), and spring migration (March 1 – April 30). The basemap was created using data from Natural Earth.
